# Protein Expression of the Microglial Marker Tmem119 Decreases in Association With Morphological Changes and Location in a Mouse Model of Traumatic Brain Injury

**DOI:** 10.3389/fncel.2022.820127

**Published:** 2022-02-10

**Authors:** Domenico Mercurio, Stefano Fumagalli, Martin K-H Schafer, Jordi Pedragosa, Lionel Dejeumen Claude Ngassam, Verena Wilhelmi, Sarah Winterberg, Anna M. Planas, Eberhard Weihe, Maria-Grazia De Simoni

**Affiliations:** ^1^Department of Neuroscience, Istituto di Ricerche Farmacologiche Mario Negri IRCCS, Milan, Italy; ^2^Institute of Anatomy and Cell Biology, University of Marburg, Marburg, Germany; ^3^Center for Mind, Brain and Behavior, University of Marburg and Justus Liebig University Giessen, Marburg, Germany; ^4^Department of Neuroscience and Experimental Therapeutics, Institute for Biomedical Research of Barcelona, Spanish National Research Council (CSIC), Barcelona, Spain; ^5^Institut d’Investigacions Biomèdiques August Pi i Sunyer, Barcelona, Spain

**Keywords:** microglia, macrophage, traumatic brain injury, Tmem119, complement C1q, neuroinflammation

## Abstract

The activation of microglia and the infiltration of macrophages are hallmarks of neuroinflammation after acute brain injuries, including traumatic brain injury (TBI). The two myeloid populations share many features in the post-injury inflammatory response, thus, being antigenically indistinguishable. Recently Tmem119, a type I transmembrane protein specifically expressed by microglia under physiological conditions, was proposed as a tool to differentiate resident microglia from blood-borne macrophages, not expressing it. However, the validity of Tmem119 as a specific marker of resident microglia in the context of acute brain injury, where microglia are activated and macrophages are recruited, needs validation. Our purpose was to investigate Tmem119 expression and distribution in relation to the morphology of brain myeloid cells present in the injured area after TBI. Mice underwent sham surgery or TBI by controlled cortical impact (CCI). Brains from sham-operated, or TBI mice, were analyzed by *in situ* hybridization to identify the cells expressing *Tmem119*, and by Western blot and quantitative immunofluorescence to measure Tmem119 protein levels in the entire brain regions and single cells. The morphology of Iba1+ myeloid cells was analyzed at different times (4 and 7 days after TBI) and several distances from the contused edge in order to associate Tmem119 expression with morphological evolution of active microglia. *In situ* hybridization indicated an increased *Tmem119* RNA along with increased microglial complement C1q activation in the contused area and surrounding regions. On the contrary, the biochemical evaluation showed a drop in Tmem119 protein levels in the same areas. The Tmem119 immunoreactivity decreased in Iba1+ myeloid cells found in the contused cortex at both time points, with the cells showing the hypertrophic ameboid morphology having no Tmem119 expression. The Tmem119 was present on ramifications of resident microglia and its presence was decreased as a consequence of microglial activation in cortical areas close to contusion. Based on the data, we conclude that the decrease of Tmem119 in reactive microglia may depend on the process of microglial activation, which involves the retracting of their branchings to acquire an ameboid shape. The Tmem119 immunoreactivity decreases in reactive microglia to similar levels than the blood-borne macrophages, thus, failing to discriminate the two myeloid populations after TBI.

## Introduction

Microglia, the resident immune cells of the central nervous system (CNS), are normally present in the healthy brain, actively surveying the surrounding microenvironment to maintain brain homeostasis (Davalos et al., 2005). They play a panoply of functions including the support to synaptic wiring during development and the monitoring of neuronal firing in the mature brain (Kim and Vellis, 2005; Salter and Beggs, 2014). Microglia have a key role in injury repair (Colonna and Butovsky, 2017) and clearance of cellular debris in diseased conditions or during aging (Pluvinage et al., 2019), contributing to restore brain homeostasis. On the contrary, microglia may also develop harmful functions, thus, driving to unwanted synaptic pruning in neurodegenerative conditions (Hong et al., 2016a,b).

Microglia share many features with blood-borne macrophages, both myeloid populations being present and actively contributing to the post-injury inflammatory response in the brain (Hanisch and Kettenmann, 2007; Ransohoff and Perry, 2009; Zanier et al., 2015a). Traumatic brain injury (TBI) represents a condition that is driving microglia activation and macrophage recruitment. Microglia readily activate and change their morphology from ramified to ameboid with an enlarged soma and retracted processes (Hanisch and Kettenmann, 2007; Zanier et al., 2015a). These morphological changes are related to the expression of novel surface antigens and to the production of mediators that build up and maintain the inflammatory response or promote injury resolution and lesion repair (Ransohoff and Cardona, 2010). Infiltrated macrophages, at variance with microglia, undergo only minor morphological changes during recruitment and activation (McWhorter et al., 2013), maintaining a round/ameboid shape. Similar to microglia, depending on the injured microenvironment, macrophages express novel/specific antigens and mediators that define their polarization state and functions (Ajami et al., 2007). In conditions of brain injury, microglia and infiltrated macrophages are hardly antigenically distinguishable. In order to assess their specific functions, early works used techniques of irradiation chimerism or parabiosis (Ajami et al., 2011; Ransohoff, 2011). Other studies used selective depletion of blood-borne macrophages (Miró-Mur et al., 2016; Perego et al., 2016; Pedragosa et al., 2018) or resident populations (Otxoa-de-Amezaga et al., 2019). Less invasive means to distinguish resident microglia from infiltrated macrophages rely on cytofluorimetry by assessing the marker CD45, which is expressed at low levels in resident cells (Martin et al., 2017; Rangaraju et al., 2018), or more recently, on the use of single-cell RNA sequencing. This latter technique has helped identify the specific transcriptional fingerprints of selective brain myeloid populations (Borst and Prinz, 2020; Olah et al., 2020; Rajan et al., 2020; Hsiao et al., 2021). However, both cytofluorimetry and single-cell transcriptomics need tissue disintegration, thus, failing to keep enough spatial information, which is certainly a key aspect to be considered when studying focal brain injuries.

The availability of a selective antigen, which is able to distinguish the resident microglia from recruited macrophages, would help characterize the roles of microglia and macrophages in brain injury progression and resolution, and remains an open challenge. Several transgenic mouse models have been developed to allow microglia identification, i.e., using fluorescent reporters under the control of Cx3cr1, Sall1, human lysozyme, or MHC class I promoters (Wieghofer et al., 2015). However, none of the models proved effective in distinguishing different subtypes of brain resident myeloid cells in healthy or diseased conditions. Microarray and bulk-RNA-seq datasets and, more recently, single-cell transcriptomics have helped to identify new fingerprints of microglia, including the transmembrane protein 119 (Tmem119), and more recently, hexosaminidase subunit beta (Hexb) (Bennett et al., 2016; Masuda et al., 2020).

The availability of a well-working histological protocol to label Tmem119 raised an early interest in this microglial antigenic marker. The Tmem119 is a type I transmembrane protein originally identified as a regulator of osteoblast differentiation (Hisa et al., 2011), which is expressed in several tissues (Kubota et al., 2002; Kanamoto et al., 2009; Mizuhashi et al., 2012, 2015). In the brain, Tmem119 is specific to microglia, and although its role is still unknown, could represent a useful tool to differentiate resident microglia from macrophages (Bennett et al., 2016). However, the validity of Tmem119, as a specific marker of resident microglia after acute brain injury, needs validation. The present study aimed at exploring Tmem119 expression by brain myeloid cells present in the injured area after TBI, using a mouse model of controlled cortical impact (CCI; Zanier et al., 2011, 2014, 2015b; Pischiutta et al., 2014). We analyzed brain sections from sham-operated or TBI mice by *in situ* hybridization to identify the spatial distribution of cells expressing *Tmem119*, and by Western blot and quantitative immunofluorescence to measure Tmem119 protein levels. Brain myeloid cells were classified according to their morphology (Zanier et al., 2015a) and analyzed at different times (4 and 7 days after TBI) and different distances from the contused edge in the cortical area pertinent to the damage.

## Materials and Methods

### Mice

Procedures involving animals and their care were conducted in conformity with institutional guidelines in compliance with national and international laws and policies (protocol 9F5F5.81, authorization n° 753/2017-PR). We used a 9-week old male (C57BL/6J WT) mice weighing 22–28 g (purchased from Charles Rivers-Italy). The protocols and details of this report are in accordance with ARRIVE guidelines (checklist provided as Supplementary Material).

### Experimental Traumatic Brain Injury

Mice were anesthetized with isoflurane inhalation (induction 5%; maintenance 2%) in an N_2_O/O_2_ (70/30%) mixture and placed in a stereotactic frame. Mice were then subjected to craniotomy followed by induction of a controlled cortical impact (CCI) brain injury as previously described (Zanier et al., 2011, 2014, 2015b; Pischiutta et al., 2014). Briefly, the injury was induced using a 3-mm diameter rigid impactor driven by a pneumatic piston rigidly mounted at an angle of 20° from the vertical plane and applied vertically to the exposed dura mater, between bregma and lambda, over the left parietotemporal cortex. We set an impactor velocity of 5 m/s and a deformation depth of 1 mm, resulting in a severe level of injury (Brody et al., 2007; De Blasio et al., 2017). The craniotomy was then covered with a cranioplasty, and the scalp sutured. Sham-operated mice received identical anesthesia and surgery without craniotomy and brain injury.

### Tissue Processing

For histological analysis, under deep anesthesia (Ketamine 20 mg + Medetomidine 0.2 mg), animals were transcardially perfused with 30 ml of phosphate buffer saline (PBS) 0.1 mol/L, pH 7.4, followed by 60 ml of chilled paraformaldehyde (4%) in PBS. The brains were carefully removed from the skull and post-fixed for 6 h at 4°C, then transferred to 30% sucrose in 0.1 mol/L phosphate buffer for 24 h until equilibration. The brains were frozen by immersion in isopentane at −45°C for 3 min before being sealed into vials and stored at −80°C until use. Coronal brain 16 (for *in situ* hybridization) or 20 μm-thick [for reverse transcription-qPCR (RT-qPCR)] cryosections were cut serially (from bregma +1.2 mm to bregma −4 mm) at 200 μm intervals. For Western blot analysis, mice were transcardially perfused with 30 mL of phosphate buffer saline (PBS) 0.1 mol/L, pH 7.4 then the brains were carefully removed from the skull and dissected into three regions ipsilateral to the lesion, namely cortex, hippocampus, and striatum, then immediately frozen on dry ice.

### RT-qPCR

Frozen paraformaldehyde-fixed (4%) fixed brain sections were collected and processed for RNA analysis from four groups (*n* = 4 brains): sham 4 and 7 days, TBI 4 and 7 days as follows. Sections were dissected into ipsilateral and contralateral halves to the TBI lesion. Total RNA was extracted from a pool of five section halves per animal using the RNAqueous™-Micro Total RNA Isolation Kit (AM1931, Invitrogen™, Carlsbad, CA, United States). Following the removal of genomic DNA by DNase digestion (740963, Macherey-Nagel, Allen Town, PA, United States), RNA was reverse-transcribed using Superscript III (Invitrogen™, Carlsbad, CA, United States) and a mix of oligo(dT) and random primer (Invitrogen™, Carlsbad, CA, United States). A quantitative real-time RT-PCR assay for *Tmem119* as gene of interest (GOI, see validation in Supplementary Figure 1), and *Hrpt1, Gapdh, and Ppia* as genes of reference (ref) was established using an SYBR Green Kit (Applied Biosystems, Waltham, MA, United States). Standard curves for each assay were established using specific primer sets for *Tmem119* (forward GTGTCTAACAGGCCCCAGAA; reverse AGCCACGTGGTATCAAGGAG, amplicon size 110 bp), *Hrpt1* (forward GGGCTTACCTCACTGCTTTCCG, reverse CGCTAATCACGACGCTGGGA, amplicon size 125 bp), *Gapdh* (forward GGTCATCCCAGAGCTGAACG; reverse TTGCTGTTGAAGTCGCAGGA, amplicon size 210), and *Ppia* (QuantiTect primer assay QT00247709, amplicon size 119). Primer amplification efficiencies were *E* = 1.980 for *Tmem119*, *E* = 1.981 for *Hrpt1, E* = *1.957 for Gapdh*, and *E* = *1.974 for Ppia.* For quantification of *Tmem119* messenger RNA (mRNA), the 2^ΔΔCT^ method was employed comparing CT values of TBI RNA samples (ΔCT[treated] with CT values of Sham controls ΔCT[control]) after normalization to the concentrations of the three reference genes *Hrpt1, Gapdh, and Ppia* by averaging their CT values {CT[ref] = mean (CT[Hprt1], CT[Gapdh], and CT[Ppia])}. The ΔCT values were calculated for each cDNA sample as ΔCT = CT*_ref_* – CT*_Tmem119_* using an amplification efficiency *E* = 2. The ΔΔCT values were calculated as ΔΔCT = ΔCT_treated_ – ΔCT_control_ and expressed as fold changes of Control (2^ΔΔCT[treated]–[control]^).

### *In situ* Hybridization

For *in situ* hybridization (ISH), frozen 4% of PFA fixed sections of mouse brain were cut in frontal orientation with 16 μm thickness on a LEICA cryostat, thaw-mounted on adhesive slides, and subjected to the hybridization procedure as described previously (Schafer et al., 1992) with some modifications. After a 5 min wash in P1 buffer (100 mM Tris–HCl, 150 mM NaCl, pH 7.5), deproteination was carried out with proteinase K (Sigma-Aldrich, Steinheim, Germany, 1 μg/ml in 100 mM Tris, 50 mM Na_2_EDTA, pH 8) for 10 min at 37°C. After an additional wash in P1 buffer, an acetylation step was performed by transfer of slides in 0.1 M triethanolamine, pH 8 (T58300, Sigma-Aldrich), and incubation in the same solution after adding 0.25% v/v acetic anhydride (320102, Sigma-Aldrich) for 10 min. Slides were washed again in P1 buffer, dehydrated in 50, 70, and 100% isopropanol, and air-dried. The RNA probes for the detection of mouse *Tmem119* and *C1qa* mRNAs were generated by *in vitro* transcription (IVT) of pGEM-T plasmids containing either a 1,002 bp or a 1,014 bp cDNA fragment of *Tmem119*, (NM_146162.2, nt. 192-11 and nt. 953-1966), or an 805 bp fragment of *C1qa* (NM_007372.2, nt. 204-1008), which were generated from neocortex cDNA of C57L/6N mice by PCR and, after confirming the sequence identity by Sanger double-stranded DNA sequencing (Microsynth Seqlab GmbH, Göttingen, Germany), subsequently incorporated into the plasmids by TA cloning according to the manufacture’s instructions (pGEM-T Vector System, A3600, Promega, Mannheim, Germany). Probes were synthesized in sense and anti-sense orientation by IVT with T7 and SP6 RNA polymerases (M0251S, M0207S, New England Biolabs ^®^, Frankfurt, Germany), respectively, using UTPαS[35S] (NEG039C001MC, PerkinElmer, Waltham, MA, United States) as a radioactive label. Probes were diluted to a final concentration of 50 × 10^4^dpm in hybridization buffer [50% formamide, 0.6 M NaCl, 10 mM Tris/HCl pH 7.4, 1 mM Na_2_EDTA, 1X Denhardt’s, 10% dextran sulfate, 100 μg/ml sonicated salmon sperm DNA, 0.05% (w/v) *E. coli* MRE600 tRNA, and 10 mM dithiothreitol (DTT)]. Of this mix, 50 μl were applied per slide and were cover-slipped. Slides were incubated in a humid chamber in the hybridization oven at 60°C for 16 h. After hybridization, slides were washed in decreasing concentrations of SSC (saline/sodium citrate) and RNase-treated in the following order: 2X SSC for 15 min, 1X SSC for 15 min, RNase solution (20 μg/ml RNase A, A3832.0500, AppliChem GmbH, Darmstadt, Germany), 1 U/ml RNase T1 (10109193001, Roche, Mannheim, Germany) at 37°C for 60 min, 1X SSC for 15 min, 0.5X SSC 15 min, 0.2X SSC at 60°C for 60 min, and 0.2X SSC for 15 min. Finally, slides were rinsed in distilled water, dehydrated in 50, 70, and 100% isopropanol, and air-dried. Slides were exposed to X-ray film (Carestream Health BioMax™ MR, Thermo Fisher Scientific, Schwerte, Germany) between 12 h and 3 days. Digital images were acquired and were processed with ImageJ (Schneider et al., 2012). For microscopic analysis, slides were coated with Ilford K5 nuclear emulsion (1355136, Harman Technology, Mobberley, United Kingdom). Following exposure times from 7 days (*C1qa*) and 14 days (*Tmem119*), autoradiograms were developed with Ilford Phenisol developer (1757635, Harman) and Ilford Hypam fixer (1758285, Harman). Sections were counterstained with methyl green (323829, Sigma-Aldrich), and cover-slipped.

### Immunofluorescence

Single and double immunofluorescence staining was performed on 4% paraformaldehyde (PFA)-fixed sections using a monoclonal rabbit anti-mouse Tmem119 (Abcam, ab209064-GR3211228, dilution 1:150) and a polyclonal guinea-pig antiserum against rat Iba1, a marker for macrophages and microglia (Synaptic Systems, 234004/2-11, dilution 1:100), as previously described with some modifications (Schafer et al., 2010). After air-drying at 40°C for 45 min, sections were incubated for 10 min in 0.4% Triton X100, followed by blocking of endogenous peroxidase activity in methanol/0.3% H_2_O_2_ for 30 min. Non-specific binding sites were blocked with 5% bovine serum albumin (BSA) in 50 mM phosphate buffered saline (PBS, pH 7.45) for 30 min, followed by an avidin-biotin blocking step (Avidin–Biotin Blocking Kit, SP-2001, Vector Laboratories, Burlingame, CA, United States). Primary antibodies were co-applied in 1% BSA/PBS and incubated at 16°C overnight followed by 2 h at 37°C. After extensive washing in distilled water, then followed by PBS, immunoreactions for the Iba1 antibody were visualized with goat anti-guinea pig secondary antibodies labeled with Alexa Fluor™ 647 (MoBiTec GmbH, Göttingen, Germany), diluted 1:200 in 1% BSA/PBS for 45 min. The primary antibody to Tmem119 was visualized by a three-step procedure. First, a biotinylated donkey anti-rabbit IgG (711-065-152, Lot. Nr. 131559, Jackson ImmunoResearch, Ely, United Kingdom) was applied for 45 min. Then, tyramide signal amplification (TSA) was carried out according to the manufacturer’s instructions (SAT700; Applied Biosystems) followed by incubation with streptavidin, Alexa Fluor™ 488 conjugate (S11223, Invitrogen) diluted 1:200 in 1% BSA/PBS for 2 h.

### Confocal Microscopy

Confocal microscopy was done using a sequential scanning mode to avoid bleed-through effects with an A1 Nikon confocal microscope. We used excitation at 488 nm for Tmem119 and 640 nm for Iba1 signals. Large view images were acquired by a 20x 0.5 NA objective, with pixel size 0.62 μm and automatically stitched with 10% overlap. Large view images served as a reference to identify the cortical regions of interest for subsequent analysis. Three-dimensional volumes sized 210 μm × 210 μm × 12 μm were acquired with a 40x 0.75 NA objective, Nyquist zoom and a 0.2 μm pixel size, and 1.13 μm step size. Digital image analysis was done using originally developed ImageJ plugins. Briefly, the image was made bidimensional by max intensity projection and channels separated. On the Iba1 channel, we applied the background subtraction with a 50 rolling, smoothed the image, and applied the unsharp mask filter to remove the background noise and increase signal contrast. The Iba1+ cells were then segmented by using a gray level cut-off kept constant throughout the images. The identified Iba1+ cells were quantified for ImageJ’s shape descriptors, i.e., cell area (μm^2^) and circularity [4π × (Area/Perimeter^2^)] (Zanier et al., 2015a). The Iba1+ cell outline was then used as a mask and applied to the Tmem119 channel. After background subtraction with a rolling 20, Tmem119 immunoreactivity within each Iba1+ outline was calculated as the mean gray value of the pixel. To calculate Tmem119 immunoreactivity in soma vs. ramifications, the Iba1+ cell outline was modified to cut all ramifications. The new outline corresponding to the soma of the cells was applied to the Tmem119 channel to calculate the mean gray value of the pixel. Ramification signal was obtained as total – soma of the mean gray value of the pixel.

### Structured Illumination Microscopy

The SIM on brain sections was done with a Nikon SIM system with a 100 × 1.49 NA oil immersion objective, managed by NIS elements software. Tissues were imaged at laser excitation of 488 (for Tmem119) and 640 nm (for Iba1) with a 3D-SIM acquisition protocol. Fourteen-bit images sized 1024 × 1024 pixels, with a single-pixel of 0.030 μm, were acquired in a gray level range of 0–4,000 to exploit the linear range of the camera (iXon ultra DU-897U, Andor) and to avoid saturation. Raw and reconstructed images were verified by the SIMcheck ImageJ plugin (Ball et al., 2015).

### Experimental Design and Statistics

Mice were randomly allocated to surgery and assigned across cages and days. To minimize variability, all surgeries were performed by the same investigator. Subsequent histological and biochemical evaluations were performed blindly by other investigators. Groups were compared by analysis of variance and *post hoc* test or with *t*-test, as indicated in each figure legend. The parametric or non-parametric test was selected after a Kolmogorov–Smirnov test or a Shapiro–Wilk test (for small-sized groups) for normality to assess whether groups met the normal distribution. GraphPad Prism 9 was used to run statistical analysis and graphic illustrations. To define the group size we referred to a previous work calculating the TBI-induced microglia ameboid transformation based on the assessment of myeloid cell circularity (Zanier et al., 2015a). Group size was defined *pre hoc* using the formula: *n* = 2σ^2^f (α, β)/Δ^2^ [SD in groups (σ) = 18.42, type 1 error α = 0.05, type II error β = 0.2, percentage difference between groups Δ = 42]. We obtained estimated numerosity of *n* = 2.92. In order to gate different Iba1+ subsets based on morphology, we set an area cut-off at 250 μm^2^ and a circularity cut-off at 0.16. Another cut-off was set at circularity 0.5 based on our previous work (Zanier et al., 2015a) that identified this population as putative infiltrated myeloid cells.

## Results

### *Tmem119* Gene Expression Increase at 7 Days After Traumatic Brain Injury in Areas Surrounding the Contusion

**FIGURE 1:**
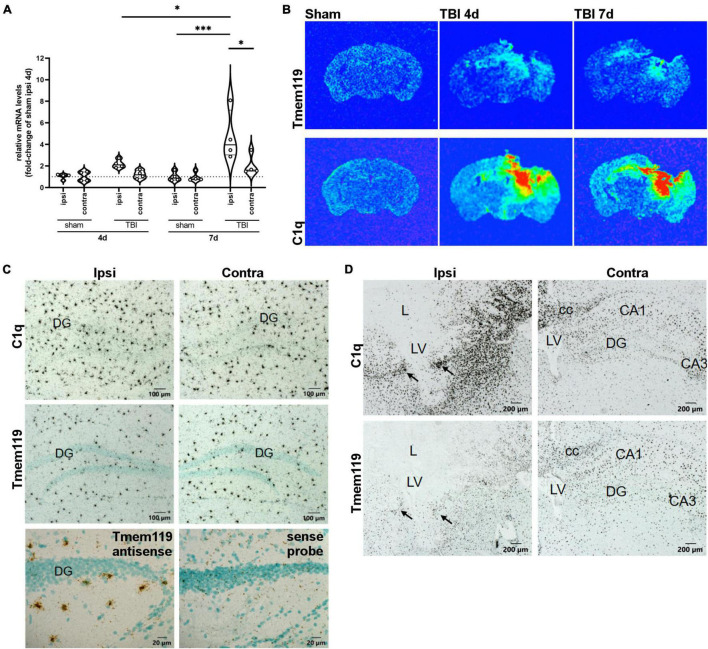
*Tmem119* gene expression at 4 or 7 days after sham surgery or TBI. **(A)** The RNA isolated from brain sections enabled comparison of *Tmem119* expression levels ipsilaterally and contra-laterally to the lesion. Data are shown as violin plot with single replicates, a line at median and quartiles, and *n* = 4. Three-way ANOVA followed by Sidak’s multiple comparison test, ^∗∗∗^*p* < 0.001, ^∗^*p* < 0.05. **(B)** Representative false color X-ray film images of coronal brain sections of TBI or sham mice after hybridization with ^35^S-Labeled RNA probes specific for *Tmem119* and *C1qa*. Exposure times of X-ray films were 48 h for *C1q*, 70 h for *Tmem119*. **(C)** High magnifications of representative autoradiograms of sham mice at 4 days. Comparing ipsi to contralateral sides, no differences were observed in the scattered microglia-like distribution pattern of *Tmem119* mRNA and *C1qa* mRNA expressing cells. Control staining with anti-sense probes produced specific *Tmem119* ISH signals in microglia, but not in neurons or other cell types. In contrast, sense probes used as negative controls produced a background but no specific cellular staining. Scale bars 100 μm or 20 μm. **(D)** High magnifications of representative autoradiograms of TBI mice at 4 days. Numerous densely packed cells in the ipsilateral areas surrounding the lesion (L) exhibit strong ISH signals for both *C1qa* and *Tmem119* (arrows). The ISH signals appeared to increase to a lesser extent also on the contralateral side. Scale bars 200 μm; cc, Corpus callosum; L, lesion area; LV, lateral ventricle; DG, dentate gyrus; and CA3, cornu ammonis region 3.

The *Tmem119* mRNA expression was quantified by RT-qPCR ipsilaterally and contra-laterally to the lesion at 4 and 7 days after sham or TBI surgery (Figure 1A). *Tmem119* mRNA levels were expressed relative to the ipsilateral hemisphere of sham mice at 4 days. *Tmem119* tended to increase about 2.2-fold in the ipsilateral cortex of TBI mice at 4 days, compared to sham mice (2.18 ± 1.9-fold-change ± SD, Figure 1A, *p* = 0.16). At 7 days, *Tmem119* expression did not change in sham mice, either in the ipsilateral or contralateral side compared to sham 4 days. However, *Tmem119* mRNA levels increased about 4.5-fold in the ipsilateral hemisphere after TBI (4.36 ± 1.57 fold-change, *p* = 0.0002) compared to 7 days TBI contra-lateral (1.92 ± 1.49 fold-change, *p* = 0.116) and 7 days sham ipsilateral (0.96 ± 1.49 fold-change, *p* = 0.0001), as well as to 4 days TBI ipsilateral (2.18 ± 1.9 fold-change, *p* = 0.317, Figure 1A).

The distribution of cells expressing *Tmem119* was assessed by *in situ* hybridization on brain sections obtained at 4 days and 7 days after TBI, or in sham mice. Figure 1B shows the areas where *Tmem119* expression was increased using false color X-ray film images. Namely, in the cortex, striatum, hippocampus, and thalamus ipsilateral to the lesion, we observed increased *Tmem119* mRNA along with increased inflammation, as depicted by the expression of *C1qa* mRNA (A-Chain of C1q), a marker of activated microglia and macrophages.

Microscopic analysis of emulsion-coated and counterstained brain sections of sham-operated mice, hybridized with *C1qa* or with *Tmem119* probes, revealed a similar scattered distribution pattern of labeled cells throughout the parenchyma typical for microglia, as illustrated for the hippocampal region in Figure 1C. The ISH signals for *Tmem119* using an antisense probe were restricted to the scattered small-sized perikaryal but were not detected in neuronal perikaryal such as the granule cells of the dentate gyrus. The RNA probes in sense strand orientation produced only background signals as shown for the *Tmem119* sense probe. In brain sections of mice subjected to TBI for 4 or 7 days, a change in both the spatial distribution and ISH signal intensity was observed for both *C1qa* and *Tmem119*, as illustrated in Figure 1D. After TBI, *C1qa* mRNA-expressing cells accumulate on the ipsilateral side in all areas surrounding the lesion core (Figure 1D). Only few *C1qa* mRNA-positive cells were present in the lesion core. On the contralateral side, *C1qa* mRNA positive cell signals increased to a lower extent, mainly in the hippocampus and in fiber bundles such as the corpus callosum. The *Tmem119* ISH revealed a similar distribution pattern after TBI on the ipsilateral side of the lesion. An increase of *Tmem119* mRNA-positive cells was observed mainly in the brain parenchyma surrounding the lesion core, like *C1q* mRNA. However, labeling intensities, as compared to *C1qa*, were much lower. Particularly, the lesion core, where *C1qa* mRNA expressing cells were still present, was almost completely devoid of *Tmem119* ISH signals with the exception of single weakly labeled cells.

### Tmem119 Protein Levels Decrease Selectively in the Injured Cortex at 4 Days After Traumatic Brain Injury

**FIGURE 2:**
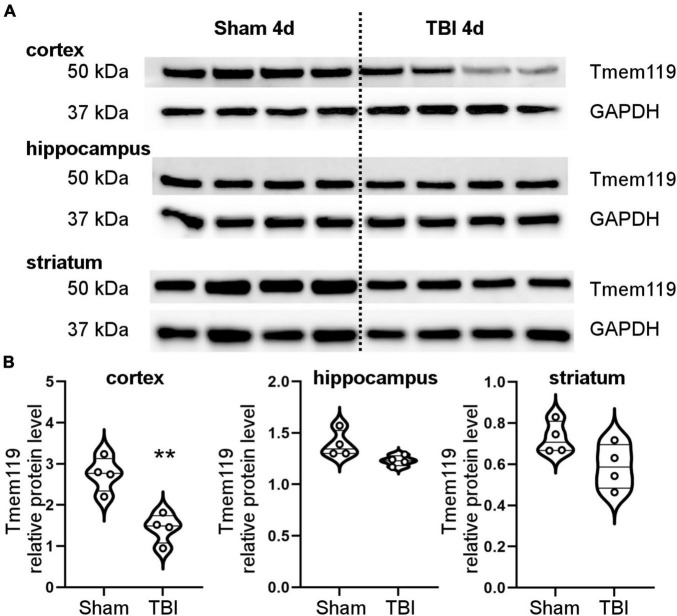
Brain protein levels of Tmem119 at 4 days after sham surgery or TBI. **(A)** Western blot showing Tmem119 protein (50 kDa) in three different brain regions ipsilateral to the lesion: cortex, hippocampus, and striatum. The GAPDH was used as a loading control. **(B)** Tmem119 protein levels decreased significantly after TBI selectively in the cortex. Data are shown as violin plot with single replicates, a line at median and quartiles, and *n* = 4. Unpaired *t*-test, **p < 0.01 vs. sham.

Brain homogenates were obtained from the ipsilateral cortex, hippocampus, or striatum of sham, and TBI mice at day 4 post-injury. We measured Tmem119 protein expression by Western blot (Figure 2A). Selectively in the cortex, Tmem119 protein levels were lower in TBI (1.44 ± 0.36 of relative protein levels ± SD) compared to sham (2.75 ± 0.42). No statistically significant differences were observed either in the hippocampus or striatum comparing TBI mice to sham mice (Figure 2B).

### Tmem119 Immunoreactivity Decrease at 4 Days and 7 Days After Traumatic Brain Injury in Cortical Areas With Strong Myeloid Cell Activation

**FIGURE 3:**
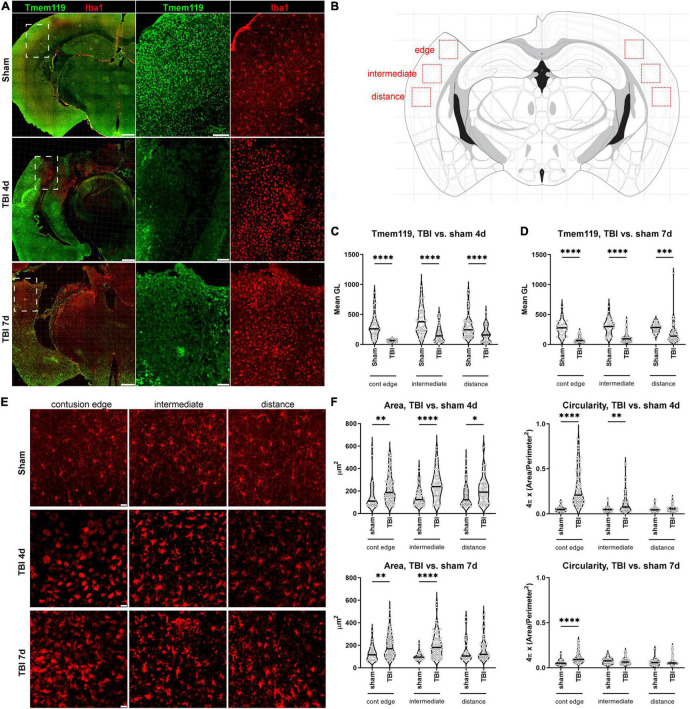
Tmem119 immunoreactivity in cortical areas with activated Iba1+ cells after TBI. **(A)** Representative large image showing Tmem119^+^ (green) and Iba1^+^ (red) cells in sham-operated and TBI mice at 4 or 7 days after injury. The Tmem119 immunoreactivity decreased after TBI and this was associated with a morphological change of myeloid cells indicative of their activation. Scale bars 500 μm in the large image, 100 μm in the magnified inserts. Inserts corresponding to white dashed boxes. **(B)** Anatomical location of the three different regions of interest (red boxes) used for subsequent Tmem119 and Iba1 quantifications. We acquired at the contusion edge and at regions located at 350 μm (intermediate) and 700 μm (distance) from it. Drawing of the section modified from the Parkinson’s atlas of the mouse brain. **(C,D)** Tmem119 levels in sham and TBI mice at 4 **(C)** and 7 **(D)** days after injury. Tmem119 immunoreactivity decreased after injury in all the areas of interest analyzed. Data are shown as violin plot with single replicates (cells), a line at median and quartiles, and *n* = 92–155 cells from three mice per group. Two-way ANOVA followed by Sidak’s multiple comparison test, ^∗∗∗^*p* < 0.01, ^∗∗∗∗^*p* < 0.0001. **(E)** Representative micro photograms showing Iba1+ cells at the three different areas of interests in sham and TBI mice at 4 and 7 days after injury. Iba1+ cells acquired an ameboid morphology when close to the contusion edge at both time points. Scale bars 20 μm. **(F)** The quantification of morphological parameters showed that Iba1+ cells had increased area and circularity, in particular, in the region near the contusion at 4 days. Data are shown as violin plot with single replicates (cells), line at median and quartiles, *n* = 92–155 cells from three mice per group. Two-way ANOVA followed by Sidak’s multiple comparison test, ^∗∗∗∗^*p* < 0.0001, ^∗∗^*p* < 0.01, and ^∗^*p* < 0.05.

Brain coronal sections were immunostained for Tmem119 and Iba1, and images were acquired by confocal microscopy. Large-view images show an even distribution of Tmem119 throughout the brain in sham mice. The Tmem119 immunostaining was detected in the typical ramified Iba1+ microglia presenting small soma (Figure 3A). Four and 7 days after TBI, areas with reduced intensity of Tmem119 immunoreactivity became apparent in the cortical area close to contusion (Figure 3A). In contrast, Iba1+ cells displayed a strongly activated morphology in the same area, i.e., hypertrophic and ameboid shapes. We quantified the Tmem119 signal in Iba1+ cells at three tissue locations in TBI mice, namely, the contusion edge, and at 350 μm (intermediate) and 700 μm (distance) from it. The corresponding areas in the sham mice, as well as in the contralateral hemisphere of TBI mice, were quantified (Figure 3B). At 4 days after TBI, the intensity of Tmem119 immunoreactivity decreased in the contusion edge (62.9 ± 19.7 of pixel’s mean gray level ± SD, Figure 3C) compared to sham (304.6 ± 192.7). A decrease of Tmem119 immunoreactivity was also observed at intermediate (174.4 ± 134.6) and distance (169.4 ± 123.4) areas in TBI compared to sham (398.5 ± 230.9 and 301.7 ± 196.4). At 7 days after TBI, Tmem119 immunoreactivity decreased at contusion edge (84.6 ± 52.1), intermediate (109.5 ± 76.9), and distant (187.9 ± 175.7) areas compared to the same regions of interest in sham mice (273.9 ± 127, 298.9 ± 133.6, 281.2 ± 72.8; Figure 3D). The same analysis done on TBI contralateral side showed a significant effect of the TBI surgery decreasing Tmem119 immunoreactivity (ANOVA surgery effect had *p* < 0.001) at 4 days, but not at 7 days (ANOVA surgery effect had *p* = 0.075, Supplementary Figure 2).

### Tmem 119 Immunoreactivity Is Lowest in Iba1+ Cells With an Ameboid Morphology

Next, we sought to see whether the decrease of Tmem119 of immunoreactivity was associated with specific morphologies of Iba1+ cells, reported to indicate specific myeloid subpopulations or activation states (Fumagalli et al., 2015). At 4 days after TBI, Iba1+ cells appeared to be mostly ameboid in the contusion edge, while ramified and hypertrophic in cortical areas located more ventrally from the edge (Figure 3E). At 7 days after TBI, most of the contusion site was lost and, thus, the edge corresponded to the forming gliotic scar (Burda and Sofroniew, 2014), where most Iba1+ cells were hypertrophic (Figure 3E). We first analyzed Iba1+ cell morphology in the cortex as previously reported (Zanier et al., 2015a). We measured cell area and circularity as indices of myeloid cell activation. At 4 days after TBI, at contusion edge, intermediate and distance regions, the cell area was increased (227 ± 127.9, 256.4 ± 129.5, and 211.4 ± 123 of μm^2^ ± SD) compared to sham (154.7 ± 116.1, 150.6 ± 85.1, and 158.3 ± 99.7). At 7 days, the cell area was still bigger at contusion edge and intermediate region (192.6 ± 100.5 and 206.1 ± 112.7) compared to sham (127.5 ± 64.5 and 99.7 ± 32.4), but not at distance (Figure 3F). Circularity was higher at contusion edge and intermediate regions (0.29 ± 0.20 and 0.12 ± 0.12 of circularity ± SD) at 4 days after TBI compared to sham (0.05 ± 0.03 and 0.05 ± 0.03). At 7 days after TBI, circularity was higher only at contusion edge (0.11 ± 0.06) compared to sham (0.06 ± 0.03, Figure 3F).

When we compared the morphology of Iba1+ cells between the sham and the hemisphere contralateral to TBI, we did not observe significant changes (Supplementary Figure 2).

**FIGURE 4:**
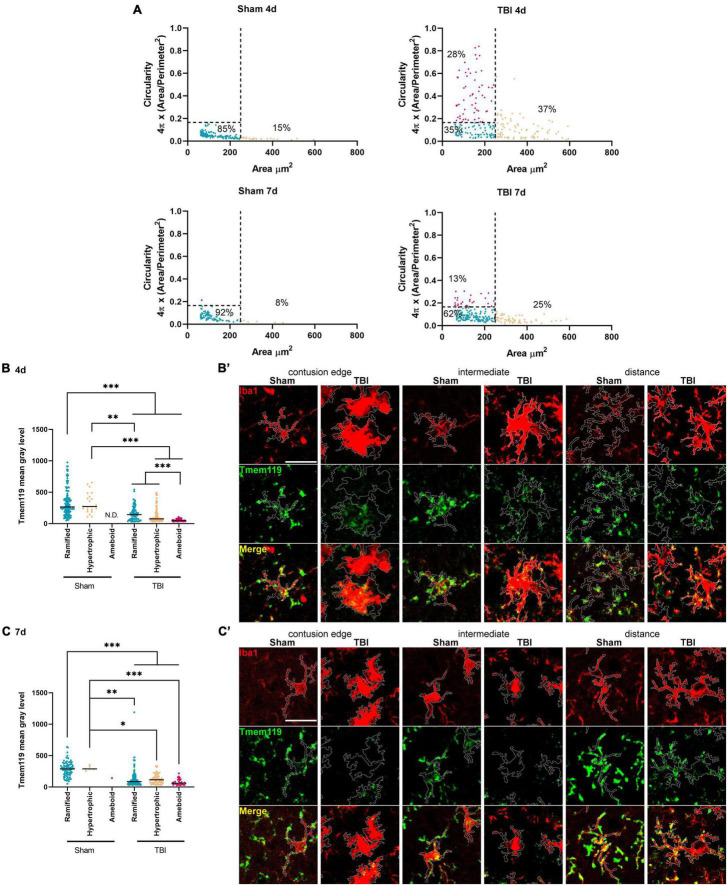
Stratification of Iba1^+^ cells based on their morphology and Tmem119 immunoreactivity after Iba1+ morphological stratification. **(A)** Distribution of Iba1+ cells based on area and circularity. We gated the cells based on the area (cut-off at 250 μm^2^) and circularity (cut-offs at 0.16). Cut-offs are indicated by the dotted lines. The relative proportions of Iba1+ in each gated population are indicated in the graph. **(B,B’)** The Tmem119 levels of each gated Iba1+ cell populations decreased significantly at 4 days after TBI compared to sham, with ameboid and cells having the lowest Tmem119 values of immunoreactivity **(B)**. Representative micro photograms showing Iba1^+^ (red) and Tmem119^+^ (green) cells in the three regions of interest. The Tmem119 immunoreactivity was calculated within the Iba1+ outline (white) traced using an originally developed ImageJ algorithm (**B’**, scale bar 20 μm). **(C,C’)** The Tmem119 immunoreactivity of each gated Iba1+ cell populations decreased significantly at 7 days after TBI compared to sham **(C)**. Representative micro photograms showing Iba1^+^ (red) and Tmem119^+^ (green) cells (**C’**, scale bar 20 μm). Data are shown as scatter dot plot, a line at mean, and *n* = 92–155 cells from three mice per group. Kruskal–Wallis test followed by Dunn’s multiple comparisons tests, ^∗∗∗^*p* < 0.001, ^∗∗^*p* < 0.01, and ^∗^*p* < 0.05.

Cell area and circularity were used to identify myeloid cells with different activation states. Based on the data obtained in the sham animals we defined, an area cut-off of 250 μm^2^ and a circularity cut-off of 0.16 were able to include 90% of all Iba1+ cells identified in sham mice (Figure 4A, defined as *ramified*). Other identified myeloid cell morphologies were *ameboid* (area <250 μm^2^ and circularity >0.16) and *hypertrophic* (area >250 μm^2^). Using these morphological cut-offs, we could observe the changes induced by TBI to the myeloid cell population composition of the cortex involved in the lesion, i.e., at 4 days after TBI 37.2% of hypertrophic, 27.5% of ameboid, with ramified decreased to 35.3%. At 7 days after TBI, we found 25.1% of hypertrophic, 12.3% of ameboid with 62.1% of ramified. At this time point, cells that we classified as *ameboid* have decreased, likely due to the loss of the contused tissue that could not be analyzed histologically.

At 4 days after TBI, all Iba1+ cell subtypes showed reduced Tmem119 immunoreactivity compared to sham, with ramified having 162.3 ± 114.1 (TBI) *vs.* 335.8 ± 218.7 (sham), hypertrophic 137.4 ± 124.8 vs. 326.7 ± 161.7 of mean gray level ± SD. The subtypes present only in TBI, i.e., ameboid cells had the lowest Tmem119 immunoreactivity (54.7 ± 18.3) when compared to the other Iba1+ cells of TBI or to sham mice (Figures 4B,B’). The measure of Tmem119 immunoreactivity based on the signal intensity may fail to identify a complete expression switch off. Indeed, we analyzed 12-bit images, having gray level values ranging from 0 to 4,096, with background noise pixels having a value >0. As such, measuring low Tmem119 immunoreactivity could not effectively distinguish between Tmem119 negative *vs.* barely positive cells, i.e., a residual gray level mean value is always measured.

At 7 days after TBI, ramified (123 ± 128.8), hypertrophic (127.1 ± 74.5), and ameboid (77 ± 48) microglia had lower Tmem119 immunoreactivity compared to sham’s ramified (285.5 ± 117.3) and ameboid cells (298.4 ± 34.1, Figures 4C,C’). At this time point, Tmem119 immunoreactivity was similar in all subtypes found in TBI mice.

### Tmem119 Protein on Thin Branches of Iba1+ Cells

**FIGURE 5:**
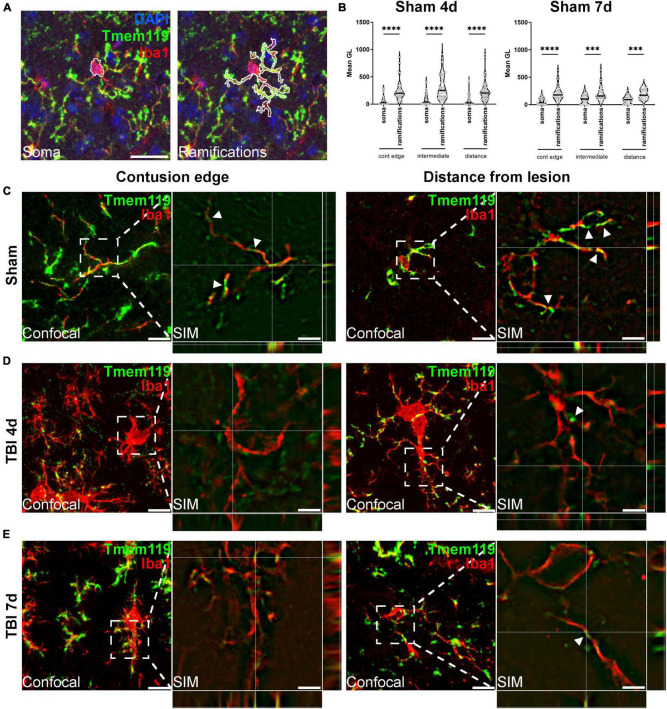
Subcellular localization of Tmem119. **(A)** Outline tracing for the differential analysis of Tmem119 in soma vs. ramifications of Iba1+ cells. Scale bar 20 μm. **(B)** Tmem119 immunoreactivity was higher in ramifications compared to the soma of Iba1+ cells at 4 and 7 days after sham surgery. Data are shown as violin plot with single replicates (cells), line at median and quartiles, *n* = 92–155 cells from three mice per group. Two-way ANOVA followed by Sidak’s multiple comparison test, ^∗∗∗^*p* < 0.001, ^∗∗∗∗^*p* < 0.0001. **(C)** We analyzed the subcellular localization of Tmem119 by super-resolved SIM. Tmem119 (green) appeared to be predominantly located on Iba1+ cell (red) ramifications, as clearly seen on the highly ramified cells present in sham mice. **(D,E)** In the contused cortex, at either 4 **(D)** and 7 **(E)** days after TBI, Tmem119 was still visible on myeloid cells ramifications of hypertrophic Iba1+ cells. Scale bars 10 μm in confocal images, 2 μm in SIM images. SIM images present z-stack orthogonal views and arrowheads indicating a few Tmem119 positive ramifications.

In order to study Tmem119 cellular localization, we further analyzed the confocal images of sham mice by differentially calculating Tmem119 immunoreactivity in soma *vs*. ramifications of Iba1+ cells (Figures 5A,B). At 4 days after sham surgery, Tmem119 appeared to be present mainly on ramifications in all the three anatomical areas analyzed (edge: 245.5 ± 180.1 *vs*. 59.1 ± 83.9 of mean gray level ± SD; intermediate: 316.8 ± 227.8 vs. 81.6 ± 111.5; and distance: 239.5 ± 176.9 *vs.* 62.2 ± 94.9). Similar results were obtained at 7 days after sham surgery (edge: 211.1 ± 136.3 vs. 62.8 ± 62.3; intermediate: 200.3 ± 133.5 vs. 98.7 ± 82.8; and distance: 191 ± 94 vs. 90.2 ± 70.2).

We then investigated Tmem119 subcellular localization using super-resolved structured illumination microscopy (SIM). Figure 5 shows the representative microphotographs obtained at the contusion edge or distance. In both areas in sham mice, Iba1+ presented Tmem119 on their ramifications, as clearly depicted using SIM (Figure 5C). Similarly, hypertrophic Iba1+ cells had Tmem119 on their ramifications after TBI (Figures 5D,E), but TBI reduced the Tmem119 expression as well as microglia ramifications. The SIM images were validated using SIMcheck (Ball et al., 2015). The results of the validation are shown in Supplementary Figure 3. The SIM achieved an actual resolution of 186 nm for Tmem119 and 182 nm for Iba1.

## Discussion

The present study shows that Tmem119 immunoreactivity decreased in activated microglia after TBI in mice. Ameboid microglia had a similar drop in Tmem119 immunoreactivity compared to sham mice, as well as to hypertrophic microglia found in the contused cortex.

This study addresses the ongoing challenge to properly characterize the distinct roles of microglia and macrophages in brain injury progression and resolution, presently hampered by the lack of selective markers (Fumagalli et al., 2015). Indeed, the frequently used markers – i.e., CD68, MHC class II, CD11b, or Iba1 – failed to distinguish between resident microglia and infiltrating macrophages (Ruan et al., 2020). Techniques like cytofluorimetry and single-cell RNA sequencing were able to classify the distinct brain myeloid populations, but do not allow the description of their micro topical localization, nor of their interactions with other cells in the brain’s microenvironment. Novel markers of microglia have been mainly identified under physiological conditions but need validation under pathological conditions like TBI.

TBI is acute damage that is driving immediate and lasting local inflammation that involves microglia activation and recruitment of macrophages. Local inflammation contributes to lesion expansion to areas surrounding the primary site of injury, and thus, offers pharmacological targets. In order to foster the research on brain myeloid cells activated in the contused area, we explored Tmem119 as a putative selective marker of microglia. The Tmem119 is a type I transmembrane protein specifically expressed by resident microglia in the healthy brain, but not by blood-borne macrophages (Bennett et al., 2016), pointing to Tmem119 as a promising selective microglial marker. However, the validity of Tmem119 as a microglia marker after TBI is yet to be confirmed. Recently, Young et al. (2021) evaluated the expression of Tmem119 to distinguish between microglia and infiltrating macrophages in a mouse model of ischemic stroke. They reported that Tmem119 presence was decreased in ramified microglia in a brain region proximal to the site of injury, thus, concluding that Tmem119 is not a stable microglia marker in the context of ischemic stroke. Of note, although microglia was reported to differ between sexes (Villa et al., 2019), Tmem119 decrease after the ischemic injury was similar in male and female mice. In line with the data on Tmem119 after the ischemic injury, we could demonstrate that the immunoreactivity of brain myeloid cells for Tmem119 has decreased in the cortical area proximal to the contusion after TBI. Data on Tmem119 immunoreactivity were anticipated by the measure of the total protein in entire brain regions using Western blot. Protein levels of the Tmem119, relative to the housekeeping protein, dropped 4 days after TBI in the cortex, but not in striatum or hippocampus, thus, indicating a local effect occurring close to the contusion site, where microglia activation and recruitment of macrophages are expected to be the highest (Karve et al., 2016; Loane and Kumar, 2016; Witcher et al., 2018, 2021). This observation is in agreement with previous works showing that Tmem119 disappears/drops with the loss of homeostatic microglia as it occurs in Alzheimer’s Disease (Keren-Shaul et al., 2017) or in multiple sclerosis (van Wageningen et al., 2019). Besides being associated with specific myeloid subpopulations, Tmem119 expression may be environment-specific, as recently reported (Bennett et al., 2018).

While we observed a drop of Tmem119 protein levels, *Tmem119* gene expression increased in the contused area, with a micro topical distribution of its overexpression, mirroring that of *C1q*, an inflammatory gene associated with microglia activation and synaptic pruning after TBI (Krukowski et al., 2018; Ciechanowska et al., 2021). Overexpression of *Tmem119* was previously reported after 4 days, and to a minor extent, 7 days after TBI in RNA extracts of the cortex, striatum, and hippocampus ipsilateral to the contusion, along with the overexpression of a wide spectrum of cellular markers for neutrophils, CD8+ T cells, astroglia, and microglia/macrophages (Ciechanowska et al., 2021). The observation that Tmem119 protein levels clearly decreased in active microglia in the TBI brain, while the mRNA increased could be due either to the impaired *Tmem119* translation, possibly due to post-transcriptional modifications causing reduced *Tmem119* transcript stability, or to the enhanced protein degradation. These possibilities remain to be investigated. Post-transcriptional modifications of mRNA stability are mechanisms of rapid and flexible coordination of different stages of inflammation (Hao and Baltimore, 2009). Factors that promote mRNA degradation can rapidly repress protein expression despite an ongoing gene transcription, allowing quick responses of inflammatory cells. This has been demonstrated, for example, in the interferon-γ-mediated response of macrophages, where the formation of the IFNγ-activated inhibitor of translation (GAIT) complex modifies a select group of mRNAs encoding inflammatory modulators (Anderson, 2010).

Using immunofluorescence, we focused on three different cortical areas located at increasing distance from the border of the lesion, showing a temporal and spatial activation gradient of brain myeloid cells. These cells showed an ameboid morphology typical of reactive microglia and recruited macrophages in the closest region to the contusion edge at 4 days and 7 days after TBI. The cells showing an ameboid shape decreased while moving further from the contusion edge and in relation to the time point (i.e., reduced ameboid cell number at 7 days after TBI). *Vice versa*, Tmem119 immunoreactivity was higher at increasing distance from the contusion edge, i.e., Tmem119 dropped in areas populated by ameboid myeloid cells. We labeled microglia and macrophages by Iba1, a pan myeloid marker that was not able to distinguish between the two cell types (Fumagalli et al., 2015). Therefore, we decided to classify Iba1 positive cells based on morphology, by considering small round-shaped cells as macrophage-like and hypertrophic cells as reactive microglia. We classified different morphologies of Iba1-expressing cells considering the cut-off values of cell circularity and the area obtained from our previous publication of characterizing a brain myeloid cell shape in TBI (Zanier et al., 2015a). The imposed cut-offs were able to identify new populations of myeloid cells after TBI that are not observed in sham mice. In particular, after TBI, we could identify small round-shaped cells (area <250 μm^2^ and circularity >0.16) corresponding to ameboid microglia and infiltrated macrophages. This morphological phenotype had the lowest values for Tmem119 immunoreactivity at 4 days after TBI. It has been reported that peripheral blood-borne macrophages, which lack Tmem119 expression (Butovsky et al., 2014; Bennett et al., 2016; Satoh et al., 2016; Zrzavy et al., 2017), or express only low levels (Bennett et al., 2018), can acquire a microglia-like phenotype (Varvel et al., 2012), including the expression of Tmem119 (Bennett et al., 2018), when transplanted into a microglia-devoid central nervous system. In agreement with the data obtained by Western blotting showing a reduced Tmem119 protein after TBI, our immunofluorescence results suggest that ameboid microglia, as well as infiltrating macrophages, have reduced the Tmem119 protein expression, at least during the first week after TBI. Whether Tmem119 expression can increase again at later stages of TBI is still an open question. In addition, we cannot exclude the possibility that blood-borne infiltrated macrophages can switch into a microglia-like phenotype at longer time points after brain injury than those explored by us, eventually expressing higher levels of Tmem119.

As a possible technical bias, we should point out that a measure for immunoreactivity based on pixel mean gray level also provides positive values for non-stained areas (i.e., background pixels). As such, it may fail to correctly identify the negative cells over those with a residual low expression of Tmem119.

The number of ameboid cells decreased at 7 days after TBI, most probably, since the core of the lesion was lost on histological sectioning and the region of interest located closest to the contusion edge could not include it. We observed a decrease of Tmem119 immunoreactivity in all the Iba1 positive cells found in the contused cortex at both time points.

The Tmem119 was mainly localized on the ramifications of Iba1+ cells, as calculated in sham mice, which is a condition in which microglia show their fully ramified morphology. Subsequently, we investigated the cellular localization of Tmem119 on myeloid cells by taking advantage of super-resolution microscopy, thus, effectively increasing the resolution of microphotographs to ≈180 nm. Super-resolution images further showed that Tmem119 was present mainly on the ramifications either in sham or TBI mice. As such, the decrease of Tmem119 protein presence in reactive microglia may depend on the process of microglial activation which involves the retracting of their branches and the acquisition of an ameboid shape (Davalos et al., 2012). The main subcellular location of Tmem119 on microglia branches could suggest a role in sensing the microenvironment, that would be switching off after activation in response to damage.

## Conclusion

Our study demonstrates that the use of Tmem119 immunoreactivity is not a useful marker of microglia after traumatic brain injury. Tmem119 could be included in the repertoire of markers used to study microglia in combination with others, like Iba1 and CD11b for the morphology or with hexosaminidase subunit beta (Hexb), this latter persistently expressed by microglia and much less by macrophages in various disease models (Masuda et al., 2020). The identification of markers selectively informing on microglia or macrophages would help define their specific role in diseased states of the brain. This appears as an important point to fully understand the local inflammatory response in view of manipulating the neuroinflammation by favoring its protective arm and limiting its toxic consequences.

## Data Availability Statement

The datasets presented in this study can be found in online repositories. The names of the repository/repositories and accession number(s) can be found below: Figshare doi: 10.6084/m9.figshare.16952875.

## Ethics Statement

The animal study was reviewed and approved by the Italian Ministry of Health (Ministero della Salute) protocol 9F5F5.81, authorization n° 753/2017-PR.

## Author Contributions

DM and SF contributed to experimental design, conducted the experiments, acquired and analyzed the data, and drafted the manuscript. JP, LN, and VW conducted the experiments and acquired and analyzed the data. MS contributed to experimental design, conducted the experiments, acquired and analyzed the data, and edited the manuscript. SW conducted the experiments. AP and EW contributed to experimental design, analyzed the data, and edited the manuscript. M-GD conceived and designed the study, supervised the study, analyzed the data, and drafted the manuscript. All authors contributed to the article and approved the submitted version.

## Conflict of Interest

The authors declare that the research was conducted in the absence of any commercial or financial relationships that could be construed as a potential conflict of interest.

## Publisher’s Note

All claims expressed in this article are solely those of the authors and do not necessarily represent those of their affiliated organizations, or those of the publisher, the editors and the reviewers. Any product that may be evaluated in this article, or claim that may be made by its manufacturer, is not guaranteed or endorsed by the publisher.
