# Supplementary material for: Protein Expression of the Microglial Marker Tmem119 Decreases in Association With Morphological Changes and Location in a Mouse Model of Traumatic Brain Injury
Source: Front Cell Neurosci. 2022 Feb 10;16:820127. doi: 10.3389/fncel.2022.820127 (PMC8866855; doi:10.3389/fncel.2022.820127)
Supplement: Supplementary file 1 [file Data_Sheet_1.pdf]

Supplementary Material

Supplementary Figures

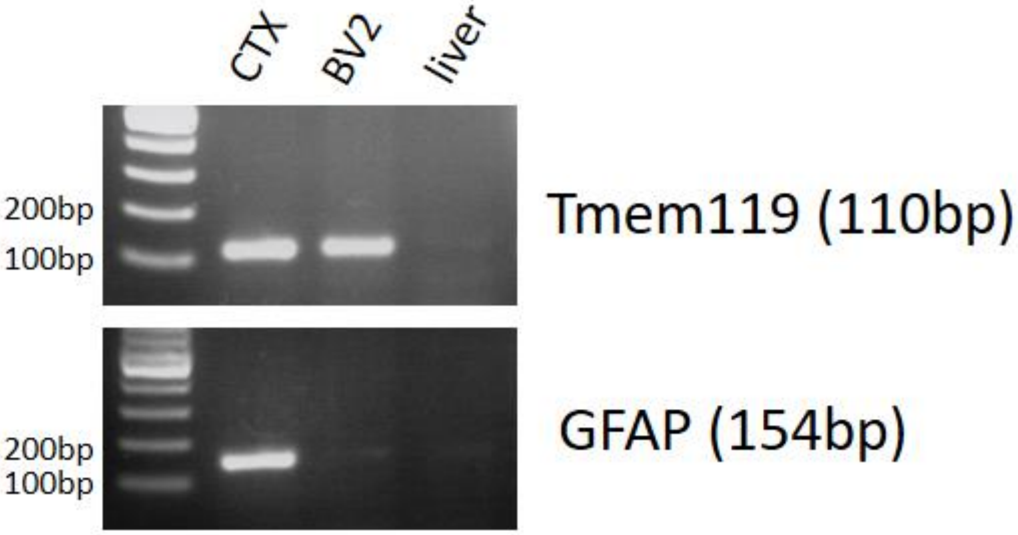

Supplementary Figure S1. Primer validation. Analysis of PCR products after RT-PCR amplification of total RNA of mouse cortex (CTX), the microglia-derived cell line BV2 and mouse liver with primer sets specific for T*mem119* (100bp) and *GFAP* (154bp).

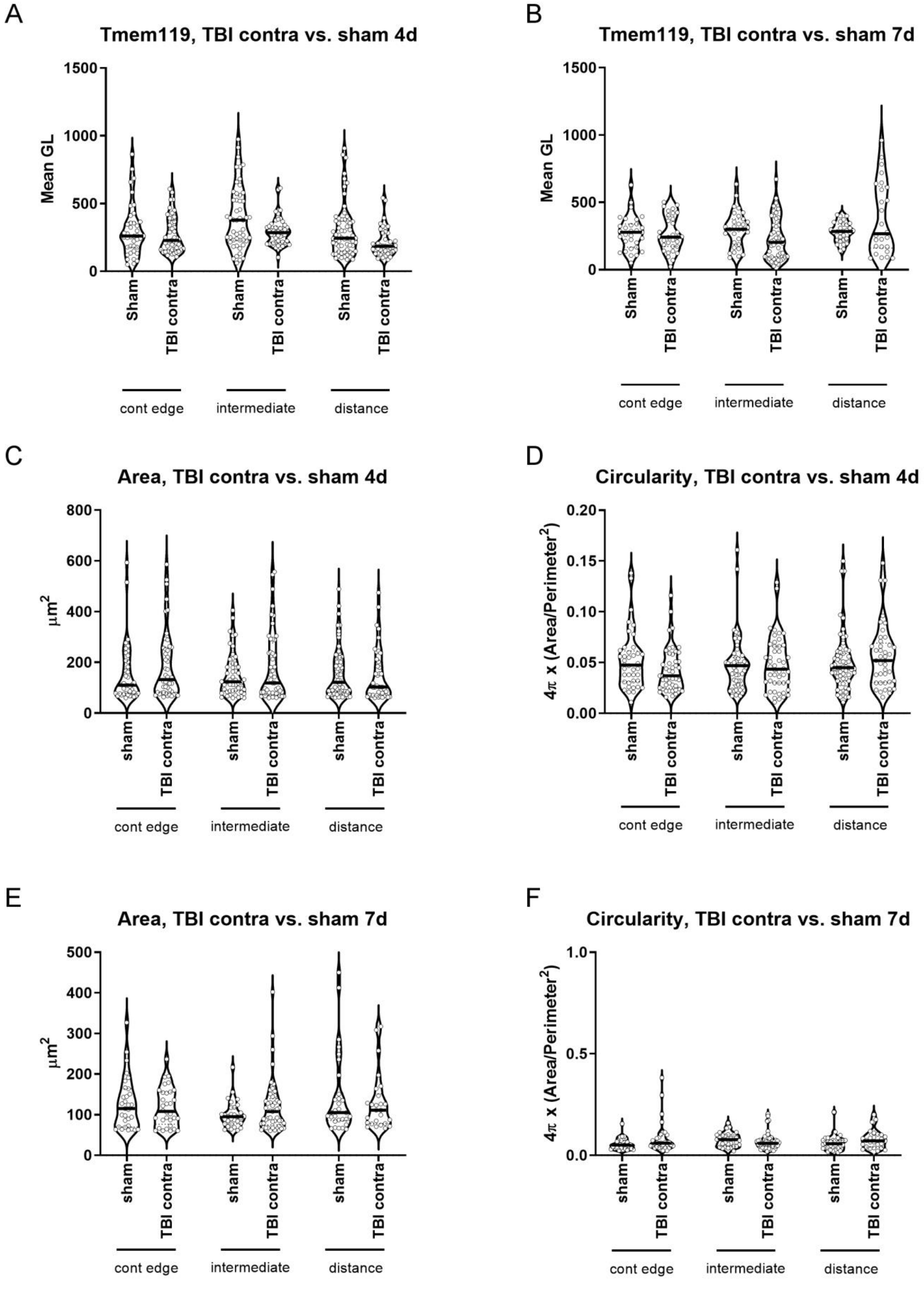

Supplementary Figure S2. Tmem119 immunoreactivity in cortical areas contra-lateral to the TBI. A-D) The quantification of morphological parameters showed that Iba1+ cells had no changes of area or circularity when comparing sham mice to the contra-lateral cortex of TBI mice at 4 (A, C) and 7 (B, D) days after injury. Data are shown as violin plot with single replicates (cells), line at median and quartiles, n= 92 –

155 cells from 3 mice per group. Two-way Anova followed by Sidak’s multiple comparison test, ns. E, F) Tmem119 levels in sham and in contra-lateral cortex of TBI mice at 4 (E) and 7 (F) days after injury did not vary. Data are shown as violin plot with single replicates (cells), line at median and quartiles, n= 92 – 155 cells from 3 mice per group. Two-way Anova followed by Sidak’s multiple comparison test

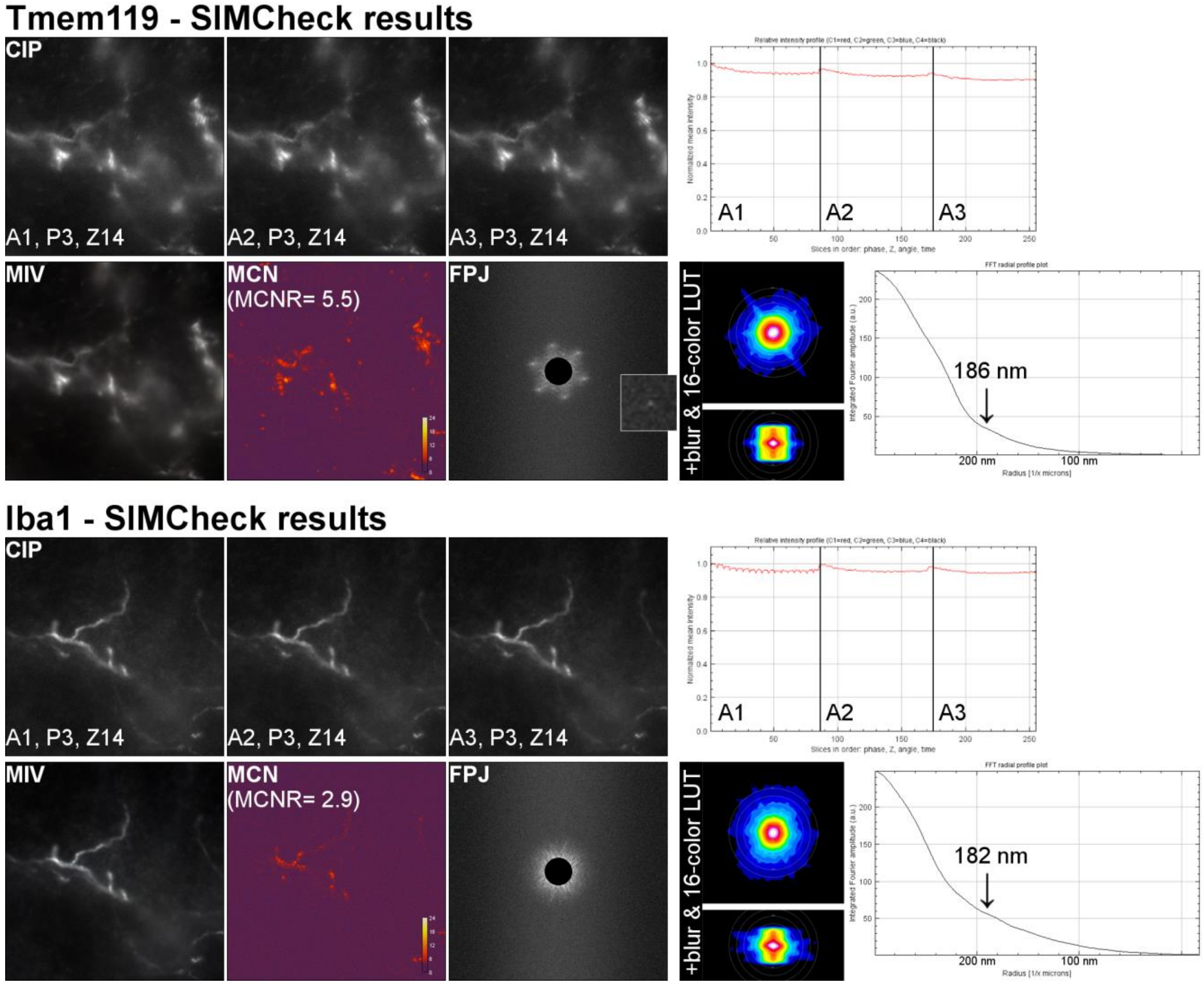

Supplementary Figure S3. SIM dataset validation by image diagnosis using SIMCheck ImageJ plugin. SIMcheck output for 3D-SIM raw dataset of Tmem119 and Iba1showing the three illumination angles at the third phase (P3) and at the 14th focal plane (Z14, **CIP**). The channel intensity profile shows limited intensity variation over phases **A1, A2** and **A3**. Motion & Illumination Variation (**MIV**) based on phase-averaged and intensity-normalized images for each angle showing a gray-white merge output, thus indicating motion stability and evennes of the illumination. Modulation Contrast-to-noise Ratio (**MCN**) showing the heatmap of local contrast which is slightly unsatisfactory only for Iba1 (<4). Raw Fourier Projection (**FPJ**) showing points of high-frequency information for each angle from first (inner spots) to second (outer spots, magnification of one spot is shown for Tmem119) order stripes. Second order spots for Iba1 are weak. SIMcheck output for 3D-SIM reconstructed dataset shows the ‘flower’ pattern in 16-color-coded image **(+blur & 16-color LUT**) for the *xy* plane and the *xz* projection. The inflection point in the radial profile plot indicates approximate effective resolution achieved in the reconstructed data (186 nm for Tmem119, 182 nm for Iba1, arrows).
